# Logistic regression analysis of textual data on suicidal ideation

**DOI:** 10.1371/journal.pmen.0000440

**Published:** 2025-09-17

**Authors:** Takafumi Kubota, Takahiro Arai

**Affiliations:** 1 School of Information and Management Sciences, Tama University, Tama, Tokyo, Japan; 2 Graduate School of Health Management, Keio University, Kanagawa, Japan; University of Canterbury, NEW ZEALAND

## Abstract

Suicide prevention requires careful consideration of the entire process, including ideation, attempt, and death. Understanding factors related to suicidal ideation is particularly critical for early intervention and effective prevention measures. In this study, we identified such factors by applying logistic regression analysis to textual data posted in September 2024 on NHK’s “Facing Suicide” website, where individuals shared their thoughts and feelings. Several keywords, including “self/identity,” were grouped into five categories, while demographic attributes such as gender, age, day of the week, and time of day were also examined. This analysis found that demographic and temporal factors influenced message content. Women and younger individuals were more likely to post messages centred on “self/identity” and “functional words/actions,” suggesting that concerns related to self-perception and existence became more pronounced during late-night hours. This temporal pattern indicates that nighttime may serve as a critical period for heightened suicidal ideation.

## Author summary

The author specializes in computational and spatial statistics, focusing on the visualization and analysis of epidemiological data—particularly related to suicide. They apply advanced statistical methods and open-source software, such as R, to construct disease maps that reveal spatial patterns in suicide mortality. This approach enables the identification of high-risk hotspots and safer coolspots, providing valuable insights into local factors influencing mental health outcomes. Beyond mapping, the author has contributed as an epidemiological expert to global health initiatives, including World Health Organization suicide reports, thereby informing prevention strategies at an international level. Their research also examines the relationship between suicide risk and temporal and lifestyle factors, comparing trends in Japan before and after the COVID-19 pandemic. By incorporating generative AI into evidence-based policy-making (EBPM), they strive to refine analytical frameworks and support informed decision-making. The author aims to drive effective interventions in future mental health policy initiatives through this blend of computational tools, spatial analysis, and public health expertise.

## Introduction

When considering suicide prevention measures, it is essential to consider the entire process that leads to suicide entirely. Specifically, there are three stages: suicidal ideation, suicide attempt, and suicide death, and the approaches and methods of data analysis required for each stage differ [1]. Suicidal ideation is the initial stage of suicidal behaviour, and understanding the causes and characteristics of suicidal ideation is extremely important for early intervention and the planning of prevention measures [2]. According to the WHO ’Live Life’ report (2021) [3], approximately one-third of individuals who die by suicide had contacted a health-care provider within the year prior to death, underscoring a critical window for early intervention. A meta-analysis by Franklin et al. (2017) [4] further indicates that the predictive accuracy of suicide-risk algorithms increases as the assessment point approaches the onset of suicidal ideation.

However, data on suicidal ideation is subjective and highly diverse, and special methods are required for analysis. In this study, we aim to clarify the factors related to suicidal ideation through logistic regression analysis using text data and to build the foundation for effective suicide prevention measures.

Randomized controlled trials of Dialectical Behavior Therapy (DBT) have shown significant reductions in suicidal ideation among adolescents after just three months of treatment (McCauley et al., 2018 [5]). Similarly, the SAFETY Program RCT demonstrated a 50% decrease in self-reported ideation at 24-week follow-up compared with treatment-as-usual (Asarnow et al., 2015 [6]).

This study aims to clarify the characteristics of the messages sent by people who are particularly struggling to live through the analysis of data related to suicidal ideation. Specifically, we will use data from the NHK website ’Facing Suicide: For a Society Where People Can Live Comfortably’ for September 2024. This data includes messages posted by people who have visited the website, in which they share their experiences and feelings, and it is also possible to obtain their demographic information (gender, age, place of residence) and the timing of their posts (day of the week, time of day, etc.). By using data from September, which includes Suicide Prevention Week in Japan, it is hoped that we will be able to gain a deeper understanding of people’s suicidal thoughts and psychological tendencies during this period.

Suicide Prevention Week in Japan is a nationally coordinated campaign held every year from 10 to 16 September, immediately following World Suicide Prevention Day (10 September). Led by the Ministry of Health, Labour and Welfare, the campaign mobilizes public agencies, mass media, and non-profit organizations to broadcast suicide-prevention messages, expand helpline capacity, and encourage the sharing of personal experiences. Television networks - including NHK - dedicate special programming and online portals during this period, resulting in a well-documented surge of user-generated content related to suicidal thoughts and help-seeking behavior. This socio-cultural context provides a unique window into ideation narratives that might otherwise remain unspoken, thereby justifying the focus on data collected in September.

There were 674 messages published during this month, of which 235 had complete attribute information. These messages contained a variety of emotions and thoughts, such as sadness and loneliness from the experience of losing someone dear and questions about oneself. Therefore, by treating these messages as text data and analyzing their frequency of occurrence and parts of speech, we could classify the thoughts and emotions of the posters into five groups. These are divided into five groups: ‘self/identity’, ‘life/existence’, ‘emotion/trial’, ‘time/frequency’, and ‘function words/behaviour’, and we will analyze how each group is related to the attributes of the message posters.

In other words, in this study, we will use logistic regression analysis for each group to statistically verify the relationship between the poster’s attributes and the messages in each group. Through this analysis, it will be possible to clarify, for example, which age groups and genders are more likely to post messages related to certain groups, and it is hoped that this will provide valuable insights as a basis for future suicide prevention activities.

Previous research on suicidal ideation has shown that a variety of factors are involved in the occurrence of suicidal ideation. Still, in many cases, analysis has been limited to specialist mental health perspectives. However, analyses based on actual data collected from the web, such as in this study, have new value in that they reflect real emotions and experiences that could not be obtained using conventional research methods. In particular, by analyzing the themes on which people with suicidal thoughts are posting and whether these differ according to demographics, it is possible to gain a deeper understanding of the diversity of individual thoughts and emotions.

Furthermore, we believe this type of analysis can also be used as evidence to help shape the direction of support measures and policies for suicide prevention. In particular, we hope that it will serve as a guideline for designing effective approaches for people who are socially isolated or who are not usually reached by support services. The study aims to contribute to the formulation of more detailed and effective suicide prevention measures than have been available to date.

## Previous studies

Research into suicidal ideation is being conducted from a variety of perspectives in Japan and overseas, and it has been shown that various factors such as age, social background and work environment have an impact. In Japan, research into suicidal ideation related to schools and universities is being actively pursued, and the need for mental health support in educational settings is being emphasized [7]. Students who are in a sensitive period of growth often experience suicidal ideation due to stress from their studies and relationships, and the importance of school support systems and preventative measures for these issues has been pointed out.

### Suicidal ideation in Japan

In particular, in recent years, methods for preventing suicide using search data on the Internet have been attracting attention. Arai et al. [8] investigated the relationship between the number of suicides and the amount of school-related Internet searches to identify search terms that could be used as leading indicators for preventing suicide among children and young people. In this study, Granger causality analysis and cross-correlation analysis were conducted on data on suicide attempts by elementary, junior high, and high school students in Japan from 2016 to 2020 and on the volume of internet searches for school-related terms on Google Trends and the temporal precedence and lag between the number of suicide deaths and the volume of searches were estimated. The results suggest that monitoring the volume of internet searches for the search term ‘I don’t want to go to school’ may be helpful for early detection of suicide risk among children and adolescents and for optimizing the display of online consultation services.

Mitsui et al. [9] aimed to predict the risk of depressive episodes and suicidal ideation among Japanese university students. Specifically, they used the Patient Health Questionnaire (PHQ-9) and the Temperament and Character Inventory and conducted multivariate logistic regression. The results showed that depressive scores and self-efficacy scores measured in the initial stage had a significant impact on the risk of subsequent depressive episodes and suicidal ideation.

In addition, as Japan’s population ages, suicidal ideation among the elderly is also seen as a serious problem. In particular, the impact of spousal loss, loneliness, and health problems on suicidal ideation among the elderly has been studied, and the need for support from local communities and welfare services has been indicated.

Noguchi et al. [10] analyzed whether social capital at the individual and community levels is related to suicidal ideation. Multilevel logistic regression analysis was conducted on the results of a questionnaire survey of residents aged 65 and over living in rural municipalities, and it was shown that a lack of trust and a lack of mutual support was associated with suicidal ideation at the individual level. In particular, for those with psychological distress, a lack of trust at the individual and community levels was significantly associated with suicidal ideation. These results showed that social capital at the individual and community levels could be a protective factor for suicidal ideation.

Awata et al. [11] evaluated the association between factors that may be related to suicidal ideation in an urban Japanese elderly population. They used data from interviews with community residents aged 70 and over in an urban area regarding suicidal ideation and socio-demographic and health-related variables. They evaluated them using univariate and multivariate logistic regression analysis. After adjusting for factors detected in the univariate analysis, depressive symptoms were strongly associated with suicidal ideation. In older adults with depressive symptoms, psychiatric disorders, including depressive and alcohol-related disorders, were significantly associated with suicidal ideation over 2 weeks. In urban communities, it is thought that screening for lack of social support, impairment of activities of daily living, and depressive symptoms, followed by diagnostic assessment of mental disorders, particularly depressive disorders and alcohol-related disorders, may be a practical and effective means of identifying older people at high risk of suicide.

### Social factors of suicidal ideation

Furthermore, it has also been noted that social factors are strongly involved in suicidal ideation, which is a characteristic of Japanese society. In particular, long working hours and interpersonal stress at work are thought to be contributing factors to suicidal ideation, and improving working environments and enhancing workplace mental health care are seen as issues that need to be addressed. In addition, social isolation, which weakens connections with family and friends, is thought to increase the risk of suicidal ideation, and there is a need for the creation of communities throughout society. In addition, demographic factors such as gender and age also have a significant impact on suicidal ideation, and there is a need for support measures tailored to each demographic group.

Aiba et al. [12] investigated several factors that affect suicidal ideation and analyzed the results of a questionnaire survey of adults on stress, stress relief, sources of social support, depressive symptoms, viewing of suicide-related websites, and suicidal ideation. The results showed that people with suicidal ideation were more likely to visit suicide-related websites than those without suicidal ideation. In other words, it was confirmed that access to suicide websites affects suicidal ideation through depression and that emotional support affects suicidal ideation through depression in women of the same age group.

Ono et al. [13] conducted face-to-face interviews with adults living in seven regions. They assessed important suicide-related outcomes and risk factors, particularly mental disorders, using the WHO Composite International Diagnostic Interview (CIDI). As a result, the risk of suicide attempts and suicide plans was highest when suicidal ideation occurred early and within one year of the onset of suicidal ideation. In addition, in the middle-aged group, the period after first employment and the presence of a mental disorder were risk factors.

### Depression and suicidal ideation

Takada et al. [14] investigated the relationship between lifestyle, work environment, depressive symptoms and suicidal ideation in order to improve the management of depression and the prevention of suicide. The measured variables included work stress factors, working hours, overtime, smoking status, alcohol consumption, sleep, exercise, diet and family factors. Using stepwise multiple logistic regression analysis, the authors examined employees from 11 cities and regions across Japan. They found that, for both men and women, exposure to strong occupational stress, problem drinking, a sense of sleep deprivation, the absence of confidant friends, and the non-use of stress coping methods were all variables that were associated with depressive symptoms. Furthermore, for both men and women, problem drinking and the absence of confidant friends were both associated with suicidal ideation.

In Sugawara et al. [15], gender differences in risk factors associated with depressive symptoms and suicidal ideation among middle-aged Japanese workers were evaluated. Data on demographic and lifestyle factors, suicidal ideation rates, the ‘Brief Job Stress Questionnaire (BJSQ)’ and the ‘Depression Symptoms Scale (CES-D)’ were collected from questionnaires administered to workers selected from companies in northern Japan. It was shown that, in men, being married, a lack of stress reduction techniques, and low job fit were independent risk factors for suicidal ideation, while in women, being married, a lack of sleep, and a lack of stress reduction techniques were significant independent risk factors.

### Suicidal ideation outside Japan

On the other hand, there are many studies of suicidal ideation outside Japan, particularly those targeting young people. For example, in Hong Kong and China, research has been conducted into the factors that contribute to suicidal ideation among young people and how family support and relationships at school affect them. By considering differences in cultural background and education systems and referring to the results of research in other countries, it has been shown that it is possible to apply the results to suicide prevention measures in Japan.

Sun et al. [16] examined a model of suicidal ideation in which family cohesion, expression, conflict, teacher support, teacher-student relationships, and peer support were used as antecedent factors, and self-esteem and depression as mediating factors. They conducted a survey questionnaire targeting Chinese adolescents in Hong Kong, collected and analyzed the data, and found that only family cohesion, conflict, teacher support, and peer support significantly predicted self-esteem and depression. In addition, it was shown that depression is a strong mediating factor in suicidal ideation.

Chang et al. [17] created a Chinese version of the Positive and Negative Suicide Ideation Inventory for junior high school and high school students in China and evaluated its psychometric properties. The scores for each subscale in the first wave of the survey were found to statistically significantly predict suicidal behaviour one year later, providing evidence of predictive validity.

As described above, existing research on suicidal ideation examines suicide risk from a variety of perspectives, including social, work environment, age and gender. It provides a basis for prevention measures and interventions. However, many of these studies are based on survey data and questionnaire results, and there are few attempts to directly analyze the raw text and expressions sent out by people who have suicidal thoughts. In particular, it can be said that the classification of words and expressions in text and the perspective of how the attributes of the people sending them (such as age, gender, and place of residence) are related to suicidal thoughts have not yet been fully elucidated.

### Logistic regression analysis of text data

This research is unique in that it uses actual text data, classifies the content into five categories (self/identity, life/existence, emotion/trial, time/frequency, function words/action), and clarifies the relationship with the poster’s attributes. Such an approach is expected to provide deep insights into suicidal ideation and new directions for prevention that cannot be obtained from conventional quantitative surveys.

Logistic regression is also considered a useful analytical method for classifying text data and analyzing high-dimensional data. It is attracting attention as a method for statistically identifying the impact of words and expressions on suicidal ideation. Logistic regression is valued for its ability to clarify the extent to which specific factors contribute to suicidal ideation while comparing the impact of various variables.

In Genkin et al. [18], a simple Bayesian logistic regression approach is applied to high-dimensional data such as natural language text, using Laplace prior distributions to avoid overfitting and generate a sparse predictive model for text data.

In addition, Taddy, M. [19] introduces a framework for dimensionality reduction of sentiment satisfaction for text data. It introduces multinomial inverse regression as a general tool for simplifying the prediction set, which can be expressed as an extraction from the multinomial distribution. It shows that low-dimensional document representations rich in sentiment information can be obtained by applying phrase count logistic regression to document annotation.

Furthermore, a chapter of logistic regression in [20] applied logistic regression to compare synonyms in corpus data, identifying various factors that affect synonym selection and distinguishing the effects of each.

However, there have been few or no studies to date that have classified messages related to suicidal ideation in detail and examined how the attributes of the posters affect each classification using logistic regression analysis. Such analytical methods are effective for elucidating the implications behind complex text data, but there are few examples of their systematic application to messages related to actual suicidal ideation. In this study, we aim to fill this gap by classifying messages related to suicidal ideation and then analyzing in detail how the attributes of the posters affect each classification. We believe that this approach will enable us to gain a deeper insight into suicidal ideation than has been possible with previous quantitative research. In addition, we expect that the analysis results will provide useful insights for suggesting new measures for suicide prevention. These results will enable more effective and targeted interventions in suicide prevention efforts.

To illustrate how timely, targeted support can translate into measurable reductions in suicidal ideation across different life stages and service settings, we cite the following three anonymised case reports drawn from publicly available sources. Case A: A 17-year-old high-school student who disclosed suicidal thoughts via an online chat service received immediate cognitive-behavioral counselling; six-week follow-up showed complete resolution of ideation (Osaka City Mental Health Report, 2022 [21]). Case B: In a workplace gatekeeper program, a 45-year-old male employee expressed suicidal ideation and was referred to onsite counselling; quarterly HR reports documented sustained absence of ideation (Ministry of Health, Labour and Welfare, 2020 [22]). Case C: During Japan’s Suicide Prevention Week, a 23-year-old university student accessed campus crisis services within 24 hours of posting self-harm intent; her SIDAS score dropped by 60% over eight weeks (Kato et al., 2019 [23]).

## Methods

### Ethics statement

As this was a study involving human subjects, the matter was discussed at the Education Research Promotion Committee of the Faculty (the School of Management and Information) at Tama University, where the author is affiliated. During this discussion, a statement that formal consent had also been confirmed, and the matter was approved (Approval No. 8).

We conducted a retrospective observational study using text data voluntarily posted on the website *’Facing Suicide: For a Society Where People Can Live Comfortably’* [24]. Specifically, we performed a logistic regression analysis in which the dependent variable was the class assigned to each post (The groups are determined as shown below.).

Although a range of high-dimensional NLP techniques—such as Latent Dirichlet Allocation (LDA), latent semantic analysis, or transformer-based representations-were available, we adopted a logistic-regression framework for three reasons. First, our theoretical aim was not to discover latent topics but to quantify how each a priori lexical group (g1-g5) relates to demographic and temporal attributes; logistic models return readily interpretable odds ratios for this purpose. Second, the final analytic sample comprised 235 posts, and a parsimonious model with few parameters mitigates the over-fitting risk that data-hungry topic or neural models often face. Third, logistic regression yields confidence intervals and significance tests that can be communicated to public-health practitioners without specialist NLP training. Topic models such as LDA would have produced clusters whose boundaries may not coincide with our predefined categories, thereby complicating the translation of findings into targeted suicide-prevention strategies.

### Data source and data preprocessing

The data used in this study was obtained from the aforementioned website [24], managed and operated by the Japan Broadcasting Corporation (NHK). This website is run as part of a joint project (the Facing Suicide Project, 2023) between NHK, the NPO Lifelink Suicide Prevention Centre, and the Japan Suicide Prevention Centre (JSCP), a general incorporated association designated by the Minister of Health, Labour and Welfare, and it provides a platform for people who are struggling with suicidal thoughts to share their experiences with the broader public. This website collects the experiences and feelings of ordinary website visitors regarding ‘difficulty in living’, and after the submitted messages have been checked, they are made public. This data is a concrete record of the loneliness, sense of loss, or worries that individuals are experiencing, and it is a valuable source of information for understanding suicidal thoughts and their background.

COVID-19 mitigation measures in Japan were officially downgraded to Class V on 8 May 2023, signalling a return to pre-pandemic social and occupational routines. To capture concerns unique to this immediate post-pandemic period, we restricted the present study to posts submitted in fiscal year 2024 and further focused on September - a month that includes Japan’s Suicide Prevention Week (10 - 16 September) - when nationwide awareness campaigns and intensified media coverage might influence help-seeking behaviour.

NHK’s public posting guidelines prohibit submissions that contain (i) graphic or violent depictions of self-harm, (ii) personal attacks or defamatory language, or (iii) excessively detailed suicide methods [25]. Accordingly, the dataset analysed here comprises only text that passed this moderation filter, and the most acute crisis narratives may therefore be under-represented.

The data includes the content each contributor has submitted as a message (hereafter referred to as ‘messages’) requested to be made public and some basic attribute information. Specifically, the data includes the messages in which the poster freely describes their own experiences and thoughts on the difficulties of living, as well as the poster’s nickname, age, sex, prefecture of residence, and date and time of posting. This attribute information provides clues to the background of each poster and helps analyze the relationship between the content of the message and the poster’s attributes. The day of the week and time of day are calculated from the date and time of posting. In addition, the entry of attribute items other than messages and nicknames is optional. The descriptions in parentheses here are variable names that will be explained in Sect 4.

As for whether or not messages are published on the website, the contributor first selects whether or not they wish to publish them, and then the administrator checks the content and makes the final decision on whether or not to publish them. This process ensures that the contributor’s privacy and ethical considerations are maintained and that reliable data is provided. The messages selected through the process are checked for appropriateness and published on the website as suitable for sharing in public. Therefore, the data used in this study is considered highly reliable because the contributors’ intentions are respected, and the administrator checks the content.

The data was collected manually from the messages and their attribute information posted on the public web pages. Specifically, we clicked the ‘read more’ button for each message to display the details and individually saved the displayed message content and attribute information. This ensured that we obtained the message content as the poster wrote it, and also collected the relevant attribute information. As a result of this data collection process, we obtained 674 messages, of which 235 had all of their attribute information recorded.

This data is not just numerical information, but conveys the feelings of the poster, such as the sense of difficulty in living or feelings of loss, and is extremely valuable in that it directly captures the individual experiences behind suicidal thoughts. Furthermore, by analyzing the combination of attribute information and message content, it is possible to clarify trends such as what kind of worries are likely to be experienced by specific age groups or genders or at what time of day suicidal thoughts are likely to intensify. In this way, it is hoped that by analyzing text data in combination with attribute data, new perspectives and strategies for suicide prevention can be found.

The data used in this study consists of 674 messages published on web pages. Data cleaning is essential to extract useful information from these data and prepare them in a format suitable for analysis. The main purpose of data cleaning is to extract frequent words based on the content of each message, organize the components of the messages, and classify them into meaningful groups.

As a first step, we extracted the most frequent words from all the messages and identified 31 keywords. This step involved analyzing the content of the messages as text data and calculating the frequency of occurrence of each word. In this extraction of the most frequent words, more than 30 words appeared with a similar frequency as the most frequent words, so we included the top 30 words, including the keywords that were ranked in the same order, and ultimately targeted 31 words. These frequent words reflect the expressions used by the contributors in their messages and the themes and emotions they emphasize, so they have an important place as the basis for analysis in this research. For full reproducibility we now supply in Appendix A (Table 4) the complete list of the 31 keywords, their Japanese orthography, romanised transliteration and group assignment.

Next, the 31 keywords were grouped together, taking into account their parts of speech and content. Pestian et al. [7], in their study of texts related to suicidal ideation (suicide-related notes), divided them into emotions, interpersonal relationships, behaviour and instructions, and other psychological states, but in this study, also taking into account that data of messages are related of ‘difficulty in living’, they added more fundamental ideas such as self and life, Each of the key words was scrutinized from a grammatical and semantic point of view. Words with similar properties were grouped as one group. As a result, the following five groups were created. The ‘self/identity’ (g1) group contains words related to self-awareness and individuals, while the ‘life/existence’ (g2) group contains words related to existence. The ‘emotion/trial’ (g3) group contained words expressing emotions and thoughts, and the ‘time/frequency’ (g4) group contained time-related expressions. Finally, the ‘function words/action’ (g5) group contained grammatical function words and words indicating actions. This classification provided a basis for analyzing the themes and perspectives of each message. The descriptions in brackets here are the variable names explained in Sect 4. Guided by suicide-related language taxonomies, we manually adapted and consolidated those frameworks into five thematic groups suitable for Japanese texts: (g1) self/identity, (g2) life/existence, (g3) relationships, (g4) future/hope, and (g5) help-seeking.

Furthermore, we checked whether or not keywords from the above five groups were used in each message. Specifically, we determined which group of the 31 words was used in each record (post). A single message could be included in a single group, multiple groups, or no groups at all. Furthermore, we checked the attributes of the messages (groups) and excluded those that included NA from the scope of this study and analysis, leaving 235 complete data sets.

Through this data cleaning, we were able to classify and organize the message content consistently and quantitatively grasp the characteristics and frequency of keywords contained in each message. The data organized in this way provides a robust foundation for clarifying the relationship between message content and the attributes of the poster in subsequent analysis.

### Five groups

The following shows the five groups classified.

g1. Self & Identity Related
  This group consists of words that indicate an individual’s identity, self-awareness, and relationships with others. Vocabulary related to the self and others reflects how an individual understands their position and how they relate to others. More specifically, words such as ‘I’, ‘myself’, ‘you’ and ‘parent’ are included, and by being used as pronouns or nouns, they express self-awareness within a context. These words, which correspond to the ‘self-category’ in cognitive linguistics, indicate roles and social positions in interpersonal relationships and reflect the self-awareness of the contributor.
g2. Life & Existence Related
  This group of words related to life, existence and daily life includes words such as ‘death’, ‘living’, ‘life’ and ‘work’. These words are essential when considering the meaning of human existence and life and are also related to basic concepts in ontology and bioethics. Expressions related to daily life and existence are used not only in a philosophical sense but also to describe the self in specific daily activities and work situations. They are essential for understanding the contributor’s attitude towards life and living.
g3. Emotion & Thought Related
  This group consists of vocabulary that expresses emotions, thoughts, and mental states. Words included are ‘feeling’, ‘painful’, ‘think’, and ‘thought’, and they reflect subjective emotions and internal thought processes. These correspond to ‘emotional categories’ in psychology and cognitive science and are essential for understanding emotions such as pain, joy, and conflict that the poster feels. When vocabulary that expresses emotions and thoughts occurs frequently, it suggests that the post is related to a specific psychological state or emotion.
g4. Time & Frequency Related
  This group of words related to time and frequency includes words such as ‘now’, ‘every day’, and ‘like this’. These words indicate the timing and continuity of events and reflect the speaker’s sense of time and daily rhythm. Expressions related to time and frequency provide clues to understanding the temporal context in which the post is written and are useful for clarifying the impact of habits and routines on suicidal ideation.
g5. Functional Words & Actions Related
  This group includes functional words that support grammatical structure and verbs that indicate specific actions. Words such as ‘say’, ‘what’ and ‘put away’ play a role in connecting the subject and predicate of a sentence or in indicating specific actions. These words clarify the semantic relationships of the whole sentence as particles or conjunctions, and as verbs, they convey particular actions. This group makes it possible to analyze the post’s grammatical structure and the action’s intention in more depth.

The five lexical groups (g1-g5) collectively capture the inherently subjective and heterogeneous nature of suicidal ideation narratives. Group 1 (“Self/Identity”) gathers personal pronouns and close-family terms (e.g., I, mother, parent), foregrounding the speaker’s inward focus. Group 2 (“Life/Existence”) contains words such as life, work, future, reflecting existential themes and questions of purpose. Group 3 (“Emotion/Thought”) comprises emotion-laden adjectives and verbs (painful, tired, cry), directly indexing the fluctuating emotional valence that characterizes ideation episodes. Group 4 (“Time/Frequency”) includes temporal adverbs like now, always, everyday, situating ideation within perceived time frames and hinting at chronicity or immediacy. Finally, Group 5 (“Functional Words/Actions”) aggregates modal and action verbs (say, do, should) that reveal intended or contemplated behaviors. Because a single post can invoke multiple groups, the taxonomy preserves the multidimensional texture of lived experience-from self-referential reflections to concrete action tendencies-thereby providing a rich, ecologically valid substrate for subsequent statistical modeling.

## Logistic regression analysis

**Fig 1:**
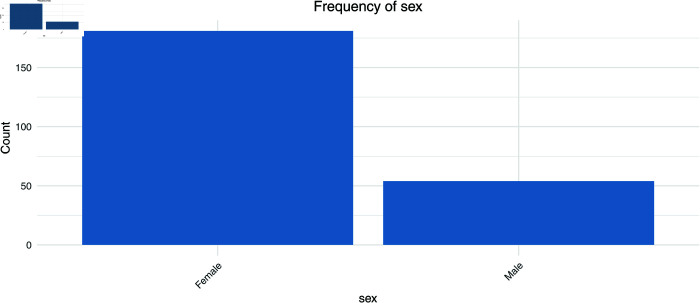
The frequency of the explanatory variables (sex).

First, Fig 1 to Fig 4 show the frequencies of the explanatory variables (in the order of gender, age, day of the week, and time).

**Fig 2:**
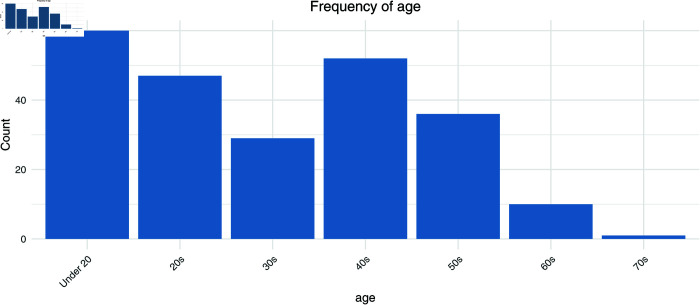
The frequency of the explanatory variables (age).

**Fig 3:**
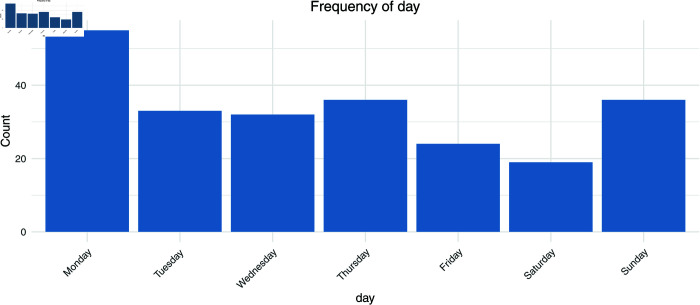
The frequency of the explanatory variables (day of the week).

**Fig 4:**
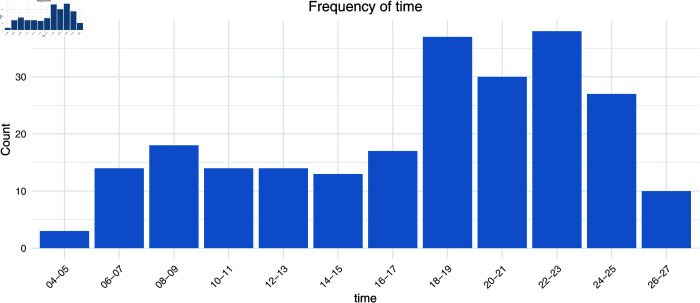
The frequency of the explanatory variables (time).

Looking at the overall trend, women account for 77.02% of the total, or around three-quarters of the total, while men account for only 22.98%. These figures suggest that the majority of message posters are women. Looking at the age groups, the proportion of people in their teens and below is exceptionally high at 25.53%, while the proportion of people in their 40s is also high at 22.13%. These stats show that the main posters are young people and middle-aged people. The 20-29 age group also accounts for a large proportion (20%), and while more than half of all users are in their 20s to 40s, the proportion of older users is extremely low, with just 0.43% in their 70s. By day of the week, Monday has the highest proportion of users at 23.4%, but Sunday also has a similar proportion to weekdays at 15.32%, showing that the site is also used at the weekend.

On the other hand, Saturday has the lowest proportion at 8.09%. Regarding the time of day, most posts are concentrated in the evening, with 16.17% being made between 10 pm and 11 pm and 15.74% between 6 pm and 7 pm. The number of posts made in the early morning hours (4 am to 5 am) is only 1.28%, showing that activity is more active during the day and into the evening. From these results, we can see that the main contributors are young to middle-aged women and that there is a tendency for posts to be concentrated in the evening. In the future, we will analyze the extremely low-frequency categories of ‘70s’ and ‘04-05’. In addition, we excluded one overseas prefecture and 14 cases where g1 to g5 were all 0 and used 221 cases for logistic regression analysis.

In this study, logistic regression analyses were conducted to examine whether participants belonged to specific groups (*g*_1_ through *g*_5_). For each model, the dependent variable was group membership (binary: 1 = belonging, 0 = not belonging), and the independent variables included sex, age, day of assessment, and time of assessment. The logistic regression model was specified as follows:


$$\log\left(\frac{P(g_{i}=1)}{1-P(g_{i}=1)}\right)=\beta_{0}+\beta_{1}\cdot sex+\beta_{2}\cdot age+\beta_{3}\cdot day+\beta_{4}\cdot time,\;(i=1,2,3,4,5)$$

To evaluate the predictive performance of each model, the following metrics were computed:

- **AUC (Area Under the Curve)**: The ROC (Receiver Operating Characteristic) curve plots the true positive rate (Recall) against the false positive rate. The AUC represents the area under the ROC curve and reflects the model’s ability to distinguish between the two classes. An AUC close to 1.0 indicates excellent discriminatory power, while an AUC close to 0.5 suggests performance no better than chance.
- **Accuracy**: The proportion of all predictions that were correctly classified.
  $$Accuracy=\frac{TP+TN}{TP+TN+FP+FN}$$
- **Precision**: The proportion of predicted positive cases that were actually positive.
  $$Precision=\frac{TP}{TP+FP}$$
- **Recall**: The proportion of actual positive cases that were correctly predicted.
  $$Recall=\frac{TP}{TP+FN}$$
- **p-Value**: The statistical significance of each explanatory variable. A *p*-value less than 0.05 was considered statistically significant.
- **True (n) and False (n)**: The number of actual cases where the outcome variable (*g*_1_ to *g*_5_) was “1” was defined as True (n), and the number of cases where it was “0” was defined as False (n):
  $$\text{True (n)}=\sum (g_{i}=1),\;\text{False (n)}=\sum (g_{i}=0)$$

A classification threshold of 0.5 was used based on predicted probabilities.

The confusion matrix terms are defined as follows:

- **TP (True Positive)**: Cases correctly predicted as belonging to the group.
- **TN (True Negative)**: Cases correctly predicted as not belonging to the group.
- **FP (False Positive)**: Cases incorrectly predicted as belonging to the group.
- **FN (False Negative)**: Cases incorrectly predicted as not belonging to the group.
- **Pseudo R**^**2**^: **McFadden’s Pseudo R**^**2**^ is a commonly used measure of model fit for logistic regression models. It is defined as:
  $$R_{\text{McF}}^{2}=1-\frac{\log L_{\text{model}}}{\log L_{\text{null}}}$$
  where $\log L_{\text{model}}$ is the log-likelihood of the fitted model, and $\log L_{\text{null}}$ is the log-likelihood of the null model (a model with only an intercept). Values closer to 1 indicate better model fit, although values around 0.2 - 0.4 are typically considered excellent for logistic regression.

All statistical analyses were conducted using R version 4.4.3.

### Summary

Table 1 shows the logistic regression analysis results for the five groups, g1 to g5, with a summary of each analysis for the 221 cases. The following points are revealed in Table 1.

**Table 1:** Logistic regression results summary.

Group	AUC	Accuracy	Precision	Recall	p-Value	True (n)	False (n)	Pseudo R^2^
g1	0.718	0.774	0.784	0.957	0.184	163	58	0.109
g2	0.699	0.828	0.827	0.994	0.543	178	43	0.095
g3	0.692	0.638	0.658	0.800	0.426	130	91	0.075
g4	0.684	0.629	0.634	0.587	0.257	109	112	0.085
g5	0.725	0.679	0.689	0.762	0.041	122	99	0.114

Note: Results include AUC, accuracy, precision, recall, p-values, sample sizes, and McFadden’s pseudo R^2^.

The area under the curve (AUC) values were 0.69 or higher, except g4. In particular, g5 (function and action-related words) had the highest value at 0.725, suggesting that the g5 model has a better predictive ability than the other groups. Regarding accuracy, g2 (life and existence-related words) had the highest value at 0.828, contributing to the accurate classification of messages. On the other hand, g4 (time and frequency related) had the lowest value of 0.629, and the models in this group had poorer classification accuracy than the others. Looking at the Precision (fitting rate), g1 (self and identity-related) had the highest value of 0.784, indicating that it had a high ability to identify messages belonging to g1 correctly is high.

Regarding Recall (reproducibility), g2 shows an extremely high value of 0.994, and almost all messages related to g2 are detected. Regarding p-value, only g5 is below the significance level (p < 0.05) at 0.041, showing a statistically significant result. In the other groups, the p-value is 0.184 or higher, and no significant difference could be confirmed.

When looking at each group individually, g2 has high Accuracy and Recall, so it is an excellent model, but the p-value is high, so it is not statistically significant. g1 has high Precision, so it can accurately classify specific messages, but it is not statistically significant overall.

In the comparison between the groups, the g5 model is the most balanced and statistically significant. These results suggest that function words and action-related keywords may be necessary in message classification.

For the McFadden’s pseudo R^2^ values, these values obtained in this study ranged approximately from **0.08 to 0.11** across all models, which corresponds to a “small effect.” This suggests that while the explanatory variables account for some of the variation in the response variable, there may be other important factors not included in the models.

Overall, the g5 model is superior to the other groups and is also statistically more reliable. In future analysis, focusing on the g5 keywords is expected to lead to more accurate message classification and a better understanding of the psychological tendencies of the posters.

### Variable importance

To assess the relative contribution of each explanatory variable in the logistic regression models, we calculated *variable importance* using the permutation importance method. This approach quantifies the impact of each variable on model performance by measuring the decrease in predictive accuracy when the values of a specific variable are randomly shuffled, thereby disrupting the relationship between that variable and the outcome.

In this study, permutation importance was computed using the vi_permute() function from the vip package in R. The target variable was the group membership outcome (*g*_1_ to *g*_5_), and the input features were sex, age, day, and time. Accuracy was used as the evaluation metric. Predictions were generated using a custom wrapper function, classifying observations as “1” if the predicted probability was greater than or equal to 0.5, and “0” otherwise.

Higher variable importance scores indicate a greater influence of the corresponding variable on model accuracy.

Table 2 also shows a list of the variable importance of the results of each logistic regression analysis. The difference in accuracy due to randomly reordering the variables is shown as the average of 1000 runs, and the P-value for testing whether the value is 0 is shown in parentheses.

**Table 2:** Variable importance results.

Group	sex	age	day	time
g1	0.039 (0.003)	0.017 (0.163)	0.027 (0.063)	0.045 (0.004)
g2	0.003 (0.467)	0.016 (0.017)	0.029 (0.016)	0.038 (0.002)
g3	0.020 (0.110)	0.056 (0.020)	0.026 (0.206)	0.023 (0.253)
g4	−0.004 (0.595)	0.032 (0.123)	0.023 (0.244)	0.078 (0.007)
g5	0.033 (0.053)	0.076 (0.001)	0.082 (0.001)	0.030 (0.084)

Note: Values in parentheses are p-values representing the statistical significance of the variable importance scores.

The results in Table 2 show a statistically significant relationship between the posters’ attributes (gender, age, day of the week, time of day) and the messages in each group.

Firstly, in g1 (self/identity), gender (importance = 0.039, p = 0.003) and time of day (importance = 0.045, p = 0.004) are significant variables. These results indicate that many women post about self and identity at specific times of day (particularly at night). Issues of self-exploration and identity are more common among women, and this tendency is more potent at night.

Next, in g2 (life/existence), age (importance = 0.016, p = 0.017), day of the week (importance = 0.029, p = 0.016), and time of day (importance = 0.038, p = 0.002) are significant. These results suggest an increase in posts related to life and existence in specific age groups (e.g. younger age groups), on particular days of the week, and at specific times of day. These results suggest a tendency to question the meaning of existence at the start of the week and night.

In g3 (emotions and thoughts), only age (importance = 0.056, p = 0.020) was significant, and the other variables were insignificant. These results suggest many posts about emotions and thoughts in specific age groups, but there is no clear relationship with gender, day of the week, or time of day. Psychological changes due to age may be affecting the content of the posts.

In g4 (time and frequency), only the time of day (importance = 0.078, p = 0.007) was significant, indicating that posts about time and frequency were concentrated in specific periods. These results reflect the tendency for thoughts about the past and future to be more active at night.

In g5 (function words and actions), age (importance = 0.076, p = 0.001) and day of the week (importance = 0.082, p = 0.001) are significant. These results show many posts related to function words and actions in specific age groups and on particular days of the week. These results indicate that posts related to behavioural change and decisions increase on particular days of the week and age.

Only g1 was significant with regard to gender, and women tended to post about self and identity. Age was significant in g2, g3, and g5, and age had an effect on message content. Day of the week was significant in g2 and g5, and there was an increase in posts of specific content on specific days of the week. Time of day was significant in g1, g2, and g4, and there was a tendency for posts on specific themes to increase at night.

The above results clearly show a clear relationship between the poster’s attributes and the message’s content. In particular, age and time of day were significant in multiple groups, indicating that the content of the posts of people with suicidal thoughts or ‘difficulty in living’ differs depending on these attributes. This finding is considered to help plan targeted support measures for suicide prevention and examine how to approach specific periods and age groups.

### Regression coefficients

For each logistic regression model, a summary table of estimated coefficients is presented. The table includes the following components:

- **Odds Ratio**: The exponentiated coefficient ($e^{\beta }$), representing the change in odds of the outcome associated with a one-unit increase in the predictor, holding all other variables constant. An odds ratio greater than 1 indicates a positive association; less than 1 indicates a negative association.
- **Coefficient**: The estimated regression coefficient (*β*), representing the change in the log-odds of the outcome for a one-unit increase in the predictor.
- **Standard Error**: The standard error of the estimated coefficient, reflecting variability in the estimate due to sampling.
- **t-value**: The ratio of the coefficient to its standard error. Larger absolute values suggest stronger evidence against the null hypothesis.
- **p-value**: The probability of observing a coefficient as extreme as the one estimated, assuming the true coefficient is zero. A *p*-value less than 0.05 was considered statistically significant.
- **Confidence Interval (CI2.5, CI97.5)**: The lower (2.5%) and upper (97.5%) bounds of the 95% confidence interval for the coefficient. If the interval does not include zero, the effect is considered statistically significant at the 5% level.

Table 3 shows a list of the regression coefficients and odds ratios for each group based on the logistic regression analysis results. This table only shows the results with a p-value of 0.1 or less.

**Table 3:** Significant variables with p-value ≤ 0.1.

Group	Variable	OR	Coef	Std. Er	t-value	p-value	CI2.5	CI97.5
g1	sex: Male	0.389	−0.945	0.388	−2.437	0.015	−1.711	−0.184
g1	day: Satur	0.162	−1.818	0.904	−2.012	0.044	−3.721	−0.109
g1	day: Thurs	0.238	−1.436	0.802	−1.790	0.074	−3.172	0.044
g2	time: 24−25	6.810	1.918	1.036	1.852	0.064	−0.035	4.170
g4	age: 30s	0.381	−0.964	0.518	−1.860	0.063	−1.278	0.560
g4	time: 08−09	5.135	1.636	0.799	2.047	0.041	0.107	3.273
g5	sex: Male	0.426	−0.854	0.385	−2.216	0.027	−1.626	−0.108
g5	age: 40s	0.226	−1.486	0.502	−2.958	0.003	−2.502	−0.524
g5	age: 50s	0.274	−1.295	0.535	−2.420	0.016	−2.371	−0.263
g5	age: 60s	0.075	−2.587	0.965	−2.682	0.007	−4.737	−0.831
g5	day: Sun	0.332	−1.102	0.639	−1.725	0.085	−2.390	0.132
g5	day: Wednes	0.275	−1.292	0.637	−2.027	0.043	−2.580	−0.065

Note: Only variables with p-value ≤ 0.1 are included. OR = Odds Ratio, Coef = Coefficient, Std. Er = Standard Error, CI2.5 and CI97.5 = 95% Confidence Interval bounds.

The results in Table 3 reveal statistically significant variables in each group.

First, in g1 (self/identity), gender and day of the week are significant. Regarding gender, the odds ratio for males is 0.389 (coefficient = −0.945, p = 0.015), indicating that males are less likely to post content that includes keywords related to self/identity than females. In terms of day of the week, Saturday (odds ratio = 0.162, p = 0.044) and Thursday (odds ratio = 0.238, p = 0.074) were significant, and there was a tendency for posts related to self/identity to decrease on these days.

Next, for g2 (life/existence), the time slot ‘24-25’ (odds ratio = 6.810, p = 0.064) was significant. These results suggest increased posts about life and existence during the late-night period. Late at night is a time when individuals reflect more deeply on themselves, and there is a possibility that they will become more active in their contemplation of the meaning of existence.

In g4 (time and frequency), the age group ‘30s’ (odds ratio = 0.381, p = 0.063) and the time slot ‘08-09’ (odds ratio = 5.135, p = 0.041) are significant. It is less likely that contributors in their 30s will make posts that include keywords related to time and frequency. On the other hand, the increase in posts containing these keywords in the morning may reflect worries or plans regarding daytime activities and how to use time.

In g5 (function words and actions), gender, age and day of the week are significant. The odds ratio for males is 0.426 (coefficient = −0.854, p = 0.027), indicating that males are less likely to post about function words and actions than females. In terms of age, the odds ratios for the 40s (odds ratio = 0.226, p = 0.003), 50s (odds ratio = 0.274, p = 0.016), and 60s (odds ratio = 0.075, p = 0.007) were significant, indicating that the older the age group, the fewer posts containing these keywords. Regarding the day of the week, Sunday (odds ratio = 0.332, p = 0.085) and Wednesday (odds ratio = 0.275, p = 0.043) were significant, indicating a tendency for posts to decrease on these days.

The above results clearly show that there is a specific relationship between the poster’s attributes and the message’s content. In particular, it is suggested that men and older people are less likely to post about self/identity, function words and actions. In comparison, women and younger people are more interested in these topics. In addition, there is a tendency for posts about specific topics to increase during late-night and morning hours, and the time of day may affect the content of the posts.

## Discussion

The results of this study revealed a significant relationship between message content and contributor attributes. In particular, the g5 (function words and action-related) model in Table 1 had the highest AUC value of 0.725, which was statistically significant (p = 0.041). However, it was not an exceptionally high value for AUC, and the model has room for improvement. However, it does show that functional words and action-related keywords play an essential role in message classification within these groups. On the other hand, g2 (life and existence) had a high accuracy of 0.828 and a high recall of 0.994, indicating high reproducibility. Still, the p-value was high, so the statistical significance was low.

In the results of Table 2, gender (variable importance = 0.039, p-value = 0.003) and time of day (variable importance = 0.045, p-value = 0. 004) were significant, indicating that there were more posts about self-identity from women and at night. In g2, age, day of the week and time of day were significant, suggesting that there were more posts about life and existence from specific age groups and late at night. These results indicate a tendency to question the meaning of existence at night and the start of the week.

The results in Table 3 also show statistically significant variables in each group. In g1, the odds ratio for males was 0.389 (p-value = 0.015), indicating that males were less likely to post about self and identity than females. In g5, it was clear that males and older age groups were less likely to post about function words and actions and that this tendency became stronger as the age group increased.

These results show that the gender, age, day of the week and time of day of the poster affect the content of the message. In particular, women and young people are more likely to post about self/identity and functional words/actions and worries about life/existence increase during the late-night period. From this, it is thought that targeted support measures that take these attributes into account are essential in suicide prevention and mental health support.

Consistent with the illustrative cases reported in the previous study section, our findings underline the need to expand night-time online chat services and workplace gatekeeper programmes, especially for adolescents and working-age adults.

Specifically, strengthening support systems during late-night hours and approaching women and young people about issues of self and identity may be effective. In addition, by reviewing support measures for men and older age groups, effective interventions can be made for groups that have previously been difficult to reach with support.

These findings are essential for understanding the relationship between the attributes of the posters and the content of their messages and provide suggestions that could lead to the development of more effective suicide prevention measures and mental health support. Specifically, they could contribute to optimizing resources at consultation services, and by identifying attributes and periods when risk is heightened, it would be possible to deploy the right personnel at the right time. These results also strengthen the necessary support for supporters who have not been adequately supported.

Suicide -related language posted on an open, self -selected web forum cannot be assumed to mirror the full spectrum of suicidal ideation that unfolds in private, offline contexts. To gauge the direction and magnitude of this sampling bias, we compared the age-sex distribution of our September dataset (*n* = 674) with the latest national suicide statistics. Whereas people aged 10–29 account for 23% of suicides nationally, they contribute 51% of the posts analysed here; women likewise are over-represented (61% vs. 37%). These discrepancies indicate that the present findings generalise primarily to younger, female internet users and should be extrapolated to other populations with caution.

### Limitations and future directions

This study has three primary limitations, the first of which concerns sample size. Although the fully-annotated analytic sample comprises 235 messages, similar - or even smaller - corpora have produced meaningful results in prior suicidality-focused NLP research; for instance, Chancellor et al. ([27]) successfully developed a risk- and protective-factor annotation scheme from just 200 posts on Reddit’s r/SuicideWatch. This precedent suggests that datasets of only a few hundred documents can still yield generalisable insights when combined with appropriate modelling and validation strategies.

A further caveat arises from the modest statistical power of our models. With only 235 fully-annotated posts, standard errors are wide and many covariate-lexical-group associations fall short of conventional significance; gender effects within the self/identity group remain the sole relationship that is both sizeable and consistently robust, whereas time-of-day for life/existence and other attribute effects display only marginal or unstable signals. Accordingly, our findings should be viewed as hypothesis-generating rather than confirmatory, and future work will require larger, longitudinal samples to verify whether the tentative patterns observed here persist and to obtain tighter confidence intervals around the weaker effects.

A second limitation concerns *content moderation* on the NHK platform. The service pre-screens submissions that contain graphic self-harm descriptions, personal attacks, or legal violations. Messages that survive this gatekeeping are therefore a *conservative slice* of crisis narratives: the most lethal plans or violent ideations may be systematically removed before reaching researchers. While we could not access the redacted corpus, we explicitly note that any risk estimates reported here are likely downward-biased. Future collaborations with platform owners will be required to quantify the scale of such removals and, where ethically permissible, include them in anonymised analyses.

A third limitation concerns on self-reported data reliability. First, the language-derived suicidal-ideation (SI) index used here should be regarded as a *proxy* rather than a replacement for established psychometric scales. Prior work that compared text-based SI signals with canonical instruments such as the Scale for Suicide Ideation and the Columbia-Suicide Severity Rating Scale has found only *moderate convergent validity* (typical Pearson r≈0.41–0.69) (O’Dea et al., 2021 [28]; Posner et al., 2011 [29]), indicating that linguistic markers capture clinically relevant variance but do not fully substitute for structured assessments.

Future studies should triangulate language data with clinician-rated interviews, objective behavioural logs (e.g., help-line utilisation, electronic health-record flags), and repeat measurements over longer intervals to establish test-retest reliability and evaluate potential method bias (Hawton et al., 2012 [30]).

Finally, the dataset rests on unverified self-reports, so future work should validate these linguistic signals against clinical assessments and other modalities (e.g., behavioural logs or sensor data).

## Concluding remarks and future studies

In this study, we analyzed the messages collected from the ‘Confronting Suicide’ website and clarified the relationship between the messages’ content and the posters’ attributes. As a result, it was shown that gender, age, day of the week and time of day affected the content of the messages. In particular, the g5 (function words and action-related) model had the highest AUC value of these and was statistically significant, but there is still room for improvement in the model. In addition, it was suggested that women and young people were more likely to post about self/identity and functional words/actions and that concerns about life/existence increased during the late-night period. These findings provide important suggestions for understanding the characteristics of people with suicidal thoughts and ‘difficulties in living’ and for developing effective suicide prevention measures and mental health support.

In future research, we will consider a method of simultaneously analyzing the keyword groups g1 to g5. These results will make it possible to clarify the interactions between each group and the complex factors involved, and we expect that it will lead to a deeper understanding of the message content. In addition, as this analysis is based on data from one month, we plan to obtain text data for one year or longer and conduct a similar study. Long-term data collection and analysis will enable us to compare results considering seasonal and temporal changes and provide more reliable findings. These efforts will make it possible to develop further new perspectives and measures for suicide prevention and mental health support.

## Appendix A: Keyword list

S1 Table lists the 31 pre-defined lexical items used in the present analysis, together with their original Japanese form, romanised transcription, group assignment, and a brief English gloss. In S1 Table, the legend descriptions for each column name are as follows. Group: Five Groups described in the Method section, Japanese: Japanese word (most frequent 31, pre-defined lexical items), Romanised: Romanised pronunciation of the Japanese word, and English gloss: Meaning of the Japanese word in English.
